# Functional Characterization and Inhibition Analysis of a Glutathione Transferase from *Cryptosporidium parvum*: A Potential Target for Antiparasitic Drug Development

**DOI:** 10.3390/ph19071106

**Published:** 2026-07-17

**Authors:** Panagiota D. Pantiora, Nikolaos D. Georgakis, Dimitris Matiadis, Marina Sagnou, Nikolaos E. Labrou

**Affiliations:** 1 Laboratory of Enzyme Technology, Department of Biotechnology, School of Applied Biology and Biotechnology, Agricultural University of Athens, 75 Iera Odos Street, 11855 Athens, Greece; pantiora@aua.gr (P.D.P.); n.georgakis@aua.gr (N.D.G.); 2 Laboratory of Chemistry, Department of Food Science & Human Nutrition, School of Food and Nutritional Sciences, Agricultural University of Athens, Iera Odos 75, 11855 Athens, Greece; dmatiadis@aua.gr; 3 Institute of Biosciences & Applications, National Centre for Scientific Research “Demokritos”, 15310 Athens, Greece; sagnou@bio.demokritos.gr; 4 Chemical Biology Laboratory, RTIN and Department of Science and Mathematics—Deree, The American College of Greece, 15342 Athens, Greece; 5 Institute of Nanobiotechnology, Sustainable Development and Artificial Intelligence, Research and Innovation Center, Agricultural University of Athens, Iera Odos 75, 11855 Athens, Greece

**Keywords:** *Cryptosporidium parvum*, glutathione transferase, enzyme inhibition, drug target, redox metabolism, curcumin analogues

## Abstract

**Background/Objectives**: Cryptosporidiosis, caused by *Cryptosporidium parvum*, is a significant cause of diarrheal disease, particularly affecting young children and immunocompromised individuals. With current treatments offering limited efficacy, there is an urgent need for novel therapeutic targets. **Methods**: In this study, we report the cloning, expression, and functional characterization of a glutathione transferase (GST) from *C. parvum* (*Cp*GST). **Results**: Biocomputing analysis revealed a single gene encoding a cytosolic enzyme with distinct structural features, compared to human cytosolic homologs. Structural modeling indicated a non-canonical thioredoxin fold and a truncated C-terminal domain, suggesting functional divergence. *Cp*GST was expressed in *Escherichia coli,* and its enzymatic properties were characterized. Although the enzyme displayed a narrow substrate spectrum, it showed a distinct substrate preference, retaining catalytic activity toward the standard GST substrates 1-chloro-2,4-dinitrobenzene (CDNB) and cumene hydroperoxide (CuOOH). Steady-state kinetic analysis revealed limited affinity for both reduced glutathione (GSH) and CDNB. Inhibition analysis identified several polyphenols and synthetic curcumin analogues as potent inhibitors, with IC_50_ values in the low micromolar range. Kinetic analysis with the most potent inhibitor revealed a mixed-type inhibition mechanism. **Conclusions**: These findings support the classification of *Cp*GST as a structurally and functionally distinct member of the GST family, likely adapted to the parasite’s physiology and metabolism. The enzyme’s divergence from human GSTs, along with its favorable druggability profile, underscores its potential as a target for anti-cryptosporidial drug development, particularly in strategies aimed at disrupting stress response and detoxification pathways.

## 1. Introduction

Cryptosporidiosis is an intestinal disease caused by intracellular protozoan parasites of the genus Cryptosporidium [1]. These parasites are a major cause of waterborne infections and outbreaks [2,3], targeting the intestinal epithelium of various hosts, including humans, livestock, birds, fish, and reptiles [4]. In humans, infection occurs primarily through ingestion of oocysts present in contaminated water or food or via direct contact with infected individuals or animals [5,6,7]. The oral–fecal route remains the predominant mode of transmission [8,9].

Cryptosporidiosis has been recognized by the World Health Organization (WHO) as one of the leading causes of parasitic gastroenteritis and diarrheal outbreaks [6]. Although it is often self-limiting in healthy individuals, the infection can become life-threatening in vulnerable populations, including young children, the elderly, and immunocompromised patients, due to prolonged and severe diarrheal episodes [10,11,12]. *Cryptosporidium parvum* and *Cryptosporidium hominis* account for up to 90% of human cases, representing a significant public health challenge [4,13,14].

Effective treatment options for cryptosporidiosis remain limited [12,15]. To date, nitazoxanide is the only drug approved by the U.S. Food and Drug Administration (FDA) for the treatment of cryptosporidiosis [16,17]. However, its efficacy is significantly reduced in immunocompromised individuals and malnourished children, populations at the highest risk of severe disease [16,17]. Other compounds, including azithromycin, paromomycin, roxithromycin, spiramycin, and bovine-derived anticryptosporidial immunoglobulins, have been explored for their therapeutic potential, but their clinical benefits remain modest [18,19,20].

Given the widespread prevalence of cryptosporidiosis in both humans and animals, the identification of novel drug targets is a critical research priority. *Cryptosporidium* species possess unique biological features that make them amenable to targeted drug development. For example, they have a compact genome and relatively simple cellular architecture, with fewer organelles compared to related protozoan parasites such as *Cyclospora*, *Plasmodium*, and *Toxoplasma* [21,22,23]. Recent studies have highlighted several promising molecular targets, particularly those involved in essential metabolic pathways and macromolecular processes. These include parasite-specific protein kinases, such as calcium-dependent protein kinase 1 (CDPK1) and phosphatidylinositol 4-kinase (PI4K) [24,25,26], as well as enzymes involved in nucleic acid synthesis [27], proteolysis (e.g., cysteine and subtilisin-like serine proteases) [28], and lipid metabolism [29].

In addition to established molecular targets, enzymes involved in the metabolism of xenobiotics and toxic compounds represent a promising yet underexplored opportunity for cryptosporidiosis drug development. Among these, glutathione transferases (GSTs) are a diverse and functionally significant superfamily of enzymes involved in detoxification, redox regulation, and xenobiotic metabolism across all domains of life [30]. They catalyze the conjugation of reduced glutathione (GSH) to a wide range of endogenous and xenobiotic electrophiles, including hydroperoxides, thereby enhancing their solubility and facilitating excretion while simultaneously protecting cells from oxidative stress [31,32]. GSTs in humans have been implicated in the development of resistance to chemotherapeutic agents and in pathogen adaptation to hostile host environments. These functional properties make GSTs attractive targets for drug development [33,34,35].

In protozoan parasites, GSTs are increasingly recognized not only for their role in cellular defense mechanisms but also as attractive drug targets, owing to their essential functions in survival and adaptation within host environments [36,37]. Among protozoa, the GSTs from *Plasmodium falciparum* and *Toxoplasma gondii* have been the most extensively studied [37,38,39]. For example, *P. falciparum* expresses a multifunctional GST involved in heme detoxification during hemoglobin digestion, a critical survival mechanism for the parasite in erythrocytes [37]. The inhibition of *P. falciparum* GST has been proposed as a viable antimalarial strategy due to its crucial involvement in oxidative stress management. Despite their well-established roles in other organisms, GSTs remain largely uncharacterized in *Cryptosporidium* species. Genomic analysis of *Cryptosporidium parvum* Iowa II by Abrahamsen et al. (2004) identified several proteins involved in the parasite’s defense mechanisms, including a single cytosolic GST [40]. This enzyme plays key roles in redox homeostasis, signal transduction, and apoptosis regulation. Importantly, it is expressed throughout all developmental stages of the Cryptosporidium life cycle, clearly suggesting its fundamental importance to parasite viability [41]. Recent genomic analyses have identified three genes encoding distinct GST isoforms in *Cryptosporidium* species [42]. Based on amino acid sequence length, these isoforms can be divided into two groups: shorter variants (157–268 residues) and longer ones (373–466 residues), with size differences attributed to the presence or absence of N- or C-terminal extensions. These GSTs are cytosolic and belong to three novel classes, Vega (θ), Psi (ψ), and Gamma (γ), which appear to be unique to *Cryptosporidium* spp. [42].

In the present study, we report for the first time the cloning and molecular characterization of a GST from the parasitic pathogen *Cryptosporidium parvum* Iowa II (*Cp*GST). Sequence analysis revealed that, although the protein shares some features with cytosolic GSTs, it exhibits substantial divergence from human GST orthologs, suggesting promising potential for selective targeting. A library of natural products, including curcumin and some of its monocarbonyl derivatives, was evaluated for their potential to inhibit the activity of *Cp*GST. This study provides the first comprehensive characterization of *Cp*GST, including its structural and functional analysis and the identification of some potent inhibitors that may act as lead therapeutic compounds.

## 2. Results and Discussion

### 2.1. Phylogenetic Analysis

Gene mining was conducted to identify putative GST genes within the *Cryptosporidium parvum* Iowa II genome (GenBank: AAEE00000000.1). The analysis led to the identification of a single open reading frame (ORF) encoding a cytosolic-type GST, which was annotated under accession number XP_628625.1 and selected for further structural and biochemical characterization. This finding should be distinguished from recent comparative genomic studies reporting three GST isoform classes across *Cryptosporidium* species, which likely reflect broader interspecies diversity within the genus. The enzyme is composed of 161 amino acids, with a theoretical pI of 4.87 and a molecular weight of 18,759.51 Da. InterPro analysis revealed that the gene encodes a protein with conserved GST structural domains, including the typical N-terminal thioredoxin-like fold (IPR004045) and a C-terminal α-helical domain (IPR010987). The obtained results indicate that the sequence contains the characteristic structural features of GSTs, thus confirming its classification within the GST family. Unlike many other protozoa that possess multiple GST isoforms, *C. parvum* appears to encode a single cytosolic GST, suggesting a potentially non-redundant and essential function. This uniqueness is in contrast to the finding in *Giardia lamblia*, where GST-like enzymes have also been identified as key antioxidant defenses, despite the organism’s limited oxidative stress response pathways [43].

**Figure 1:**
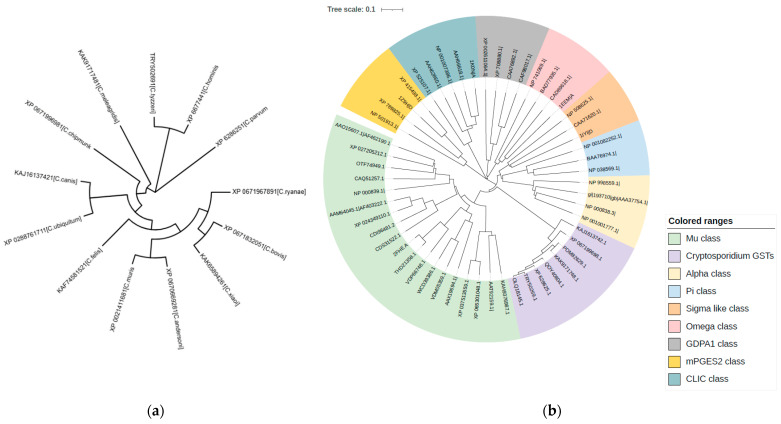
Phylogenetic analysis of *Cp*GST. (**a**) Phylogenetic tree of *Cp*GST homologs from *Cryptosporidium* species. The tree was constructed based on protein sequence alignments produced by ClustalO to illustrate evolutionary relationships among homologs from multiple *Cryptosporidium* species. Labels include GenBank accession numbers followed by species names. (**b**) Phylogenetic analysis of *Cp*GST. The *Cp*GST sequence with representative homologues from all known animal classes of GSTs. ClustalO was used for tree construction and illustrated using iTOL v5.

A BLASTp search using the *Cp*GST sequence identified homologous GST-like proteins across various *Cryptosporidium* species. The retrieved sequences exhibited pairwise identity values ranging from 55.97% for *C. muris* to 98.76% for *C. hominis*, while the corresponding query coverage values ranged from 94% to 100%. The high query coverage observed for most homologues indicates that the similarity extends across a substantial portion of the *Cp*GST sequence, supporting moderate to high conservation of GST-like proteins within the genus. These sequences were subsequently used to construct a phylogenetic tree, enabling the analysis of the evolutionary positioning of *Cp*GST (XP_628625.1) in relation to its homologs from other *Cryptosporidium* species (Figure 1a).

*Cp*GST clusters most closely with the enzyme from *C. hominis*. This supports the well-established genetic similarity between *C. parvum* and *C. hominis*, both of which infect humans and share similar biological and pathogenic features. The evolutionary analysis of GSTs from various *Cryptosporidium* species, using representative sequences from all known animal GST classes, revealed that *Cp*GST clusters closely with the mammalian mu GST class, yet it forms a distinct and separate clade. This phylogenetic positioning suggests that *Cp*GSTs probably represent a novel class of animal GSTs, diverging early from the canonical mu-type lineage (Figure 1b).

### 2.2. Structure Prediction and Analysis of CpGST

**Figure 2:**
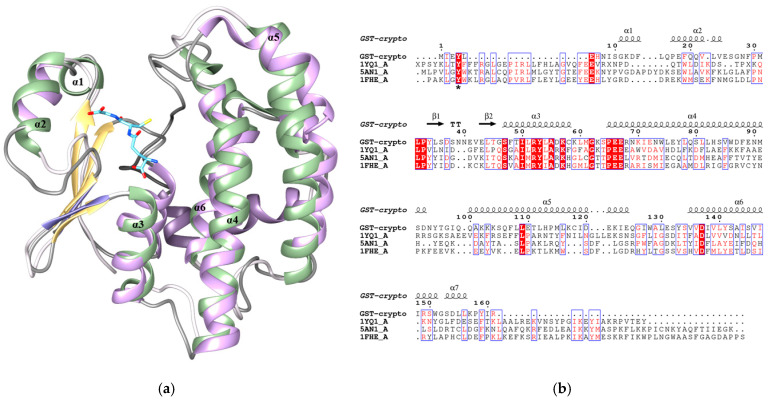
(**a**) Structural superposition of *Cp*GST with the mu-class GST from *Fasciola hepatica* in complex with GSH (PDB ID: 1FHE). *Cp*GST is shown in light green, *F. hepatica* GST is depicted in purple, and the bound GSH molecule is represented in light blue. (**b**) Multiple sequence alignment of *Cp*GST with structurally and functionally characterized GSTs. The aligned sequences include 1FHE_A (*Fasciola hepatica*, 45.45% identity), 5AN1_A (*Penaeus vannamei*, 37.18% identity), and 1YQ1_A (*Caenorhabditis elegans*, 26.36% identity). The predicted catalytic residue (Tyr4) is marked with a star (★). The alignment highlights conserved structural features and residues relevant to enzymatic function.

Structure prediction of *Cp*GST was performed using the I-TASSER server. Among the five models generated, the one with the highest confidence score was selected for further analysis. Overall, the predicted structure (Figure 2a) is characterized by the typical GST architecture, consisting of two distinct domains. The N-terminal thioredoxin-like domain, spanning residues 1 to 62, harbors the glutathione-binding site (G-site), and a C-terminal domain is responsible for binding hydrophobic xenobiotic substrates (H-site). Interestingly, although cytosolic GSTs generally exhibit the canonical βαβαββα thioredoxin-like fold, *Cp*GST displays a non-canonical thioredoxin fold with an ααββα motif, suggesting potential structural and functional divergence. The C-terminal all-alpha domain is located between residues 64 and 161. This domain is significantly shorter compared to that of other parasite and human orthologs. The C-terminal helix of GSTs plays a pivotal role in maintaining the structural integrity of the active site and shaping the hydrophobic substrate-binding pocket (H-site). It contributes to substrate orientation, active-site closure, and catalytic efficiency, often participating in induced-fit conformational changes upon ligand binding [44,45].

To increase confidence in the predicted architecture, the *Cp*GST model was further compared with AlphaFold2-derived predictions and evaluated using structural quality and confidence metrics (see Supplementary Information Figure S1a). AlphaFold2 confidence analysis showed that the predicted *Cp*GST structure has generally good to very high local confidence across most of the sequence. The five ranked models displayed highly similar pLDDT profiles, indicating good consistency among the independent predictions (Figure S1b). The best-ranked *Cp*GST model showed a mean pLDDT value of 88.87, with an uncertainty of 2.57, supporting very good overall confidence in the predicted structure. The overall agreement between independent modeling approaches supports the proposed fold.

All these features together—the structural divergences, the non-canonical thioredoxin fold and the missing C-terminal helix compared to cytosolic GSTs—make the parasite-specific *Cp*GST a compelling candidate drug target, offering opportunities for the design of selective inhibitors that minimize off-target effects on host (human) GSTs.

Structure-based alignments (Figure 2b) with mu-class GST homologs from *Fasciola hepatica* (45.45% identity), *Penaeus vannamei* (37.18% identity), and *Caenorhabditis elegans* (26.36% identity) revealed the presence of a conserved catalytic tyrosine residue at position 4 (Tyr4) [46,47]. Interestingly, in *F. hepatica*, Gln66 and Lys44 are key contributors to GSH binding [46]. In contrast, the corresponding residues in *Cp*GST is occupied by Gly46, and no Lys residue is present. These substitutions highlight the low degree of homology between the two enzymes at the G-site, which may in turn contribute to a reduced affinity for GSH.

### 2.3. Cloning, Heterologous Expression and Purification

**Figure 3:**
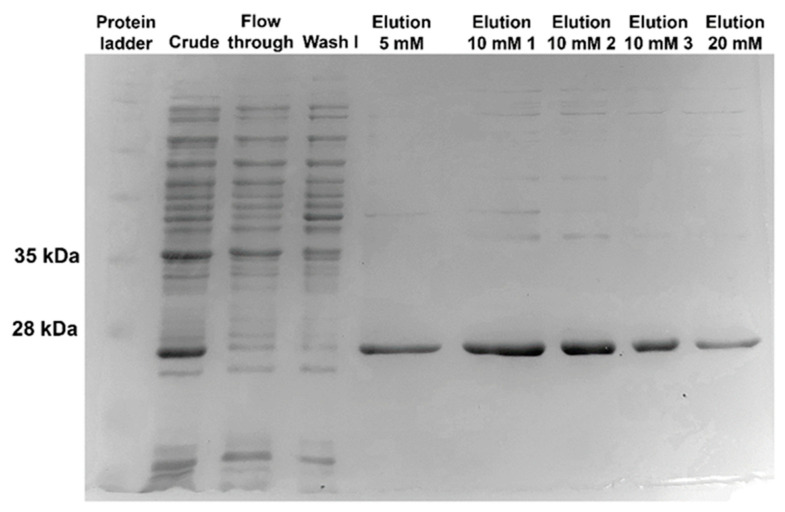
SDS–PAGE (12% *w*/*v*) analysis of *Cp*GST purification using Ni^2+^-IDA-Sepharose affinity chromatography. Lane annotations are as follows: Protein ladder (molecular weight markers), crude lysate, flow-through, wash I, and elution fractions collected with increasing imidazole concentrations (5 mM, 10 mM, and 20 mM).

The synthetic gene encoding *Cp*GST was cloned into the pET-28b(+) plasmid vector under the control of a T7 promoter, allowing for strong IPTG-inducible expression. The expression construct was transformed into *E. coli* Rosetta™ 2 (DE3) pLysS cells. Following induction with IPTG, the recombinant *Cp*GST was efficiently expressed and subsequently purified using Ni^2+^-IDA-Sepharose affinity chromatography (Figure 3). The SDS–PAGE analysis of the purification process showed a distinct band consistent with the expected molecular weight of the His-tagged *Cp*GST protein. The protein was eluted using a stepwise imidazole gradient (5 to 20 mM). This elution profile suggests a low affinity of the His-tagged *Cp*GST for the Ni^2+^ resin. Following purification, the enzyme preparation exhibited a specific activity of 0.431 U/mg, corresponding to an enzyme recovery of 81.8%. The purification protocol resulted in a 45.4-fold increase in specific activity compared with the crude extract.

### 2.4. Substrate Specificity and Kinetic Analysis

The substrate specificity of *Cp*GST was assessed by measuring its specific activity against a panel of common GST substrates (Table 1). Using CDNB, a standard model substrate for GSTs, the enzyme exhibited the highest specific activity, confirming its suitability for baseline enzymatic characterization.

Notably, *Cp*GST also showed activity toward CuOOH, a known marker of GSH peroxidase-like activity, suggesting a potential role in peroxide detoxification and redox homeostasis. In contrast, the enzyme exhibited minimal activity toward tert-butyl hydroperoxide, indicating a narrow substrate range for hydroperoxides and a possible structural preference for aromatic over aliphatic hydroperoxides. Fluorodifen, a substituted diphenyl ether herbicide, was accepted as a substrate with moderate activity, which is consistent with GSTs involved in xenobiotic metabolism. Interestingly, no detectable activity was observed toward several other known GST substrates, including dehydroascorbate, ethacrynic acid, and isothiocyanates (allyl isothiocyanate and phenethyl isothiocyanate). The inability to metabolize ethacrynic acid and isothiocyanates, substrates commonly conjugated by human GSTs [48], further supports the structural divergence of *Cp*GST from host homologs and emphasizes its unique substrate recognition profile.

Collectively, these findings suggest that *Cp*GST is catalytically active and has a restricted but distinct substrate range. Its preference for CDNB and CuOOH highlights its dual potential in both xenobiotic conjugation and peroxidase-like activity, while its inactivity toward several common GST substrates underscores its structural and functional specificity.

**Figure 4:**
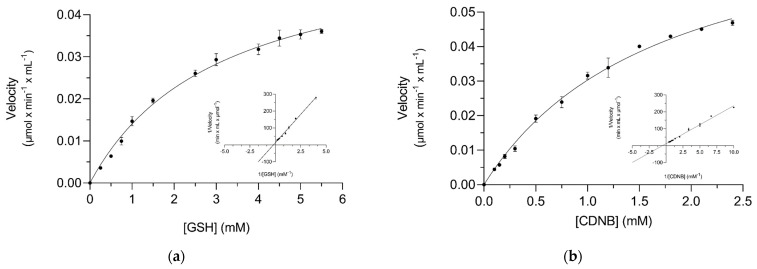
Kinetic analysis of *Cp*GST. (**a**) GSH was used as the variable substrate at a fixed CDNB concentration (1 mM), and (**b**) CDNB was used as the variable substrate at a fixed GSH concentration (2.5 mM). Enzyme activity was monitored at 340 nm, and initial velocities were calculated and fitted to the Michaelis–Menten equation. Insets show the corresponding Lineweaver–Burk double-reciprocal plots.

The kinetic parameters of the recombinant *Cp*GST enzyme were determined using GSH and CDNB. The results for the kinetic analysis are shown in Figure 4 and the related kinetic parameters are summarized in Table 2. The enzyme displayed Michaelis–Menten-type kinetics for both substrates. The high K_m_ value suggests a relatively low affinity of *Cp*GST for GSH compared to many human GSTs, which often have K_m_ values for GSH below 1 mM [49]. This observation may reflect structural divergence in the GSH-binding site, as supported by the sequence alignment and structural modeling data (Figure 2). The lower K_m_ for CDNB, compared to GSH, indicates a somewhat higher affinity for this electrophilic substrate relative to GSH, though both affinities remain in the low millimolar range. This kinetic profile aligns with the enzyme’s modest specific activity values and further supports its classification as a catalytically active but relatively low-efficiency GST compared to canonical human GSTs [49,50,51].

The low catalytic activity and substrate affinity are likely attributable to the absence of the C-terminal helix, which plays a key role in active-site stabilization and substrate positioning. In several GST classes, residues within this region directly influence substrate specificity and enzyme stability. Multiple studies have demonstrated that removal or alteration of the C-terminal helix profoundly impairs GST function. For example, studies on alpha- and theta-class GSTs have shown that removal of this region alters substrate specificity, reduces activity and disrupts proper active-site architecture [44,45]. The relatively low catalytic activity of *Cp*GST may be partly associated with its predicted structural divergence from canonical cytosolic GSTs, including the shortened C-terminal region. The purified recombinant enzyme was soluble, migrated at the expected molecular weight, and showed reproducible activity toward CDNB and CuOOH, following Michaelis–Menten kinetics. These observations support the conclusion that *Cp*GST is catalytically competent and unlikely to be misfolded. Nevertheless, other factors, including subtle conformational effects of heterologous expression, parasite-specific molecular partners, or preference for physiological substrates not tested here, may also contribute to its low catalytic efficiency. Further structural and functional studies will be required to clarify the molecular basis of *Cp*GST activity.

These results suggest that *Cp*GST may not serve a broad detoxification role under basal conditions but could instead be adapted for specific or inducible metabolic tasks within the parasite’s metabolic framework.

### 2.5. Inhibition Analysis of Polyphenols and Synthetic Curcumin Analogues Against CpGST

**Figure 5:**
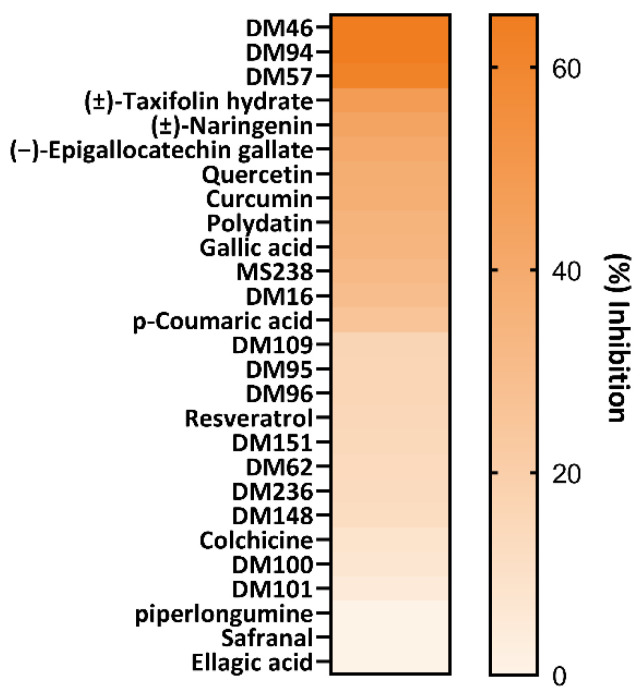
Heatmap illustrating the percentage inhibition of *Cp*GST enzyme activity by selected natural products and curcumin analogues at a concentration of 25 μM. Inhibitory potency is color-coded from low (light) to high (dark orange), as indicated by the scale bar on the right.

Inhibition analysis was conducted to assess the inhibitory potency of a range of natural products, mainly polyphenols and synthetic curcumin analogues, against *Cp*GST. Each compound was tested at a final concentration of 25 μM. The results were visualized as a heatmap (Figure 5) and listed in Table 3. The screening revealed distinct trends in inhibitor structural preferences, suggesting that certain chemical scaffolds and substituents may be critical for enzyme binding and inhibition. Among the tested natural compounds, polyphenolic flavonoids and catechins demonstrated the highest inhibitory activity. Notably, taxifolin hydrate (47.6%), naringenin (43.5%), and epigallocatechin gallate (40.7%) showed strong inhibition (>40%), suggesting that the flavonoid scaffold, particularly with multiple hydroxyl groups, enhances binding affinity to the enzyme.

Quercetin (38.1%) and curcumin (37.6%) followed closely, reinforcing the importance of planar, aromatic structures with conjugated systems and phenolic hydroxyls that can potentially engage in hydrogen bonding or π–π stacking within the active site. Gallic acid (34.3%) and polydatin (34.7%) also exhibited moderate inhibition, which may be related to their trihydroxybenzene cores, albeit with a structurally simpler architecture compared to that of flavonoids. In contrast, p-coumaric acid (25.8%) and resveratrol (15.4%) were less potent and were suboptimal for effective binding. Interestingly, colchicine (8.7%), safranal, piperlongumine, and ellagic acid showed negligible or no inhibition.

Among the most effective inhibitors identified was curcumin (37.6%). The effects of curcumin on the infectivity and development of *C. parvum* have been studied in an in vitro system combining infection of human ileocecal adenocarcinoma cell cultures with quantification of intracellular parasites by quantitative polymerase chain reaction. Curcumin exhibited >95% inhibition of parasite growth at 50 μM for 24 h when infected cultures were exposed for more than 12 h [52]. The anti-cryptosporidial effect of curcumin was also demonstrated in a study by Asadpour et al., which reported a significant reduction in oocyst shedding and intestinal lesions in both the jejunum and ileum of mice treated with either curcumin or paromomycin [53]. Interestingly, the strong antioxidant effect of curcumin was postulated to be at least partially responsible for its therapeutic mechanism in a number of studies [53,54,55]. The inhibition of phospholipase A2 and histone deacetylase family enzymes reported in *C. parvum* is an additional postulated mode of the anti-cryptosporidial action of curcumin [56,57]. To the best of our knowledge, this is the first time that this activity has been related to the inhibitory potency of curcumin against *Cp*GST.

Consequently, these results prompted us to assess the inhibitory potency of several monocarbonyl curcumin analogues against the enzyme. More specifically, the synthetic analogues displayed a broad range of inhibitory effects, highlighting the impact of substituent type, position, and hydrophobicity. DM46 (65.2%), DM94 (64.9%), and DM57 (62.3%) were the most potent inhibitors, surpassing the parent curcumin compound. Moderate inhibitors included DM16 (29.5%), MS238 (31.6%), and DM109 (16.3%). The majority of the analogues, including DM62, DM95, DM96, DM151 and DM236, exhibited only low inhibition (≤15%), and DM101 and DM100 were among the least active (≤7%), suggesting that even slight structural changes strongly affect enzyme binding.

**Figure 6:**
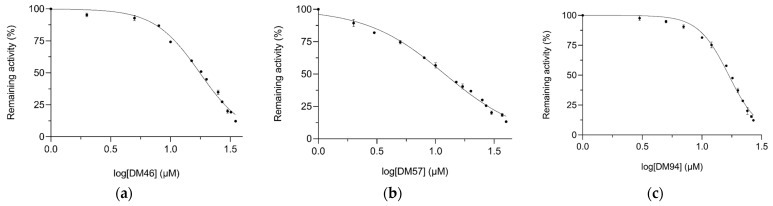
Concentration–response curves for the determination of IC_50_ values of curcumin analogues DM46 (**a**), DM57 (**b**), and DM94 (**c**) against *Cp*GST enzyme activity. Remaining enzymatic activity (%) was measured after incubation with increasing concentrations of each inhibitor. Data represent the mean ± standard deviation (SD) of three independent experiments.

Although more compounds should have been screened to establish a clearer structure–activity relationship profile, some features may still be identified as crucial for activity. The current inhibitor panel provides a useful starting point for *Cp*GST inhibitor discovery, while a larger and more structurally diverse compound library will be required in future studies to establish a robust SAR and to identify more selective and potent inhibitors. In particular, the presence of methoxy groups at various substitution positions and numbers may be identified as a highly favourable structural characteristic. This is supported not only by the fact that all three highest-activity inhibitors—DM46, DM57 and DM94—possess one, two or three methoxy groups, respectively, on each of the symmetrical aromatic rings but also by the activity of the remaining methoxylated compounds. The moderate activity for DM16 and MS238 may similarly be connected to the presence of methoxy substitution, regardless of the monocarbonyl or dicarbonyl nature of the curcuminoid skeleton. In contrast, most of the phenolic compounds exhibit rather low inhibitory potential. Additionally, the low activity of DM236 could be interpreted as an indication of the importance of the monocarbonyl moiety for effective inhibition. It is noteworthy that in our ongoing investigation for the inhibitory potential of this type of molecule against various human GSTs, like P1-1 and A4-4, the phenolic motif appeared to be more active [58]. This may provide further support for the structural divergence in the GSH-binding site. Based on these results, DM46, DM57, and DM94 were selected for further dose–response analysis to determine their IC_50_ values (Figure 6). As summarized in Table 4, DM57 emerged as the most potent inhibitor, with an IC_50_ value of 11.8 ± 0.5 μM, followed by DM94 (16.8 ± 0.5 μM) and DM46 (17.6 ± 0.6 μM).

Although the applied workflow follows a standard inhibitor-screening approach, it provides a necessary first step toward the identification of novel *Cp*GST-targeting compounds and establishes a basis for future structure-guided optimization and more advanced functional studies.

Previous work with human GSTP1-1 [31], the most widely distributed and therapeutically relevant GST isoenzyme, showed that the two most active *Cp*GST inhibitors, DM57 and DM46, exhibited only modest inhibitory activity against GSTP1-1, reducing enzyme activity by 16.75% and 8.5%, respectively [31]. Conversely, DM96 and DM100, which were weak inhibitors of *Cp*GST, showing 7.2% and 15.53% inhibition, respectively (Table 3), displayed strong inhibitory activity against human GSTP1-1, with inhibition values of 86.99% and 40%, respectively. These findings suggest a degree of selectivity of DM57 and DM46 toward *Cp*GST over human GSTP1-1, although this conclusion should be interpreted with caution, as the compounds have not yet been evaluated against the full panel of human GST isoenzymes.

**Figure 7:**
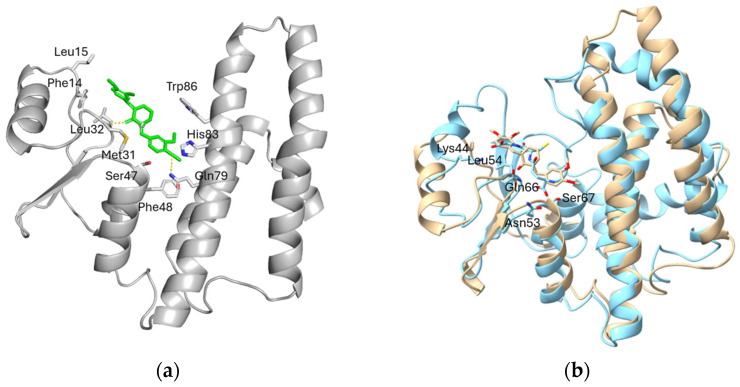
Molecular docking analysis of DM57 with *Cp*GST. (**a**) Predicted binding mode of DM57 within the AlphaFold-modeled structure of *Cp*GST. DM57 is shown in green, and amino acid residues predicted to participate in ligand binding are labelled. The binding cavity is mainly formed by hydrophobic residues, including Phe14, Leu15, Met31, Leu32, Phe48, and Trp86, together with polar residues such as Ser47, Gln79, and His83 that may contribute to ligand stabilization. Yellow dashed lines indicate predicted polar interactions. (**b**) Structural superposition of the *Cp*GST–DM57 docking model with the mu-class GST from Fasciola hepatica in complex with GSH (PDB ID: 1FHE). *Cp*GST and DM57 are shown in light brown, while *F. hepatica* GST and bound GSH are shown in light blue. Residues involved in GSH binding in *F. hepatica* GST are labelled. The comparison indicates that DM57 binds close to, but not identically within, the canonical GSH-binding region, supporting a possible non-canonical binding mode in *Cp*GST.

Molecular docking was performed to predict the best ligand binding site and explore the putative binding mode of DM57 within the predicted *Cp*GST structure (Figure 7). The docking model indicated that DM57 is accommodated in a binding cavity formed mainly by hydrophobic residues, including Phe14, Leu15, Met31, Leu32, Phe48, and Trp86, together with polar or potentially hydrogen-bonding residues such as Ser47, Gln79, and His83. The aromatic and methoxy-substituted scaffold of DM57 appears to be stabilized predominantly by hydrophobic contacts and π-related interactions with aromatic residues, particularly Phe14, Phe48, and Trp86. In addition, possible polar interactions involving Ser47, Gln79, and/or His83 may further contribute to ligand stabilization. Overall, the predicted pocket displays a mainly hydrophobic character, with localized polar residues capable of supporting specific ligand orientation.

Structural superposition of the *Cp*GST–DM57 docking model with the mu-class GST from *Fasciola hepatica* in complex with GSH (Figure 7) showed that the predicted DM57-binding region is located close to the GSH-binding region. This supports the hypothesis that DM57 may inhibit *Cp*GST through binding to a partially overlapping pocket near the substrate-binding region.

### 2.6. Kinetic Inhibition Analysis of CpGST by DM94 and Molecular Docking Assessment of Its Binding Mode

**Figure 8:**
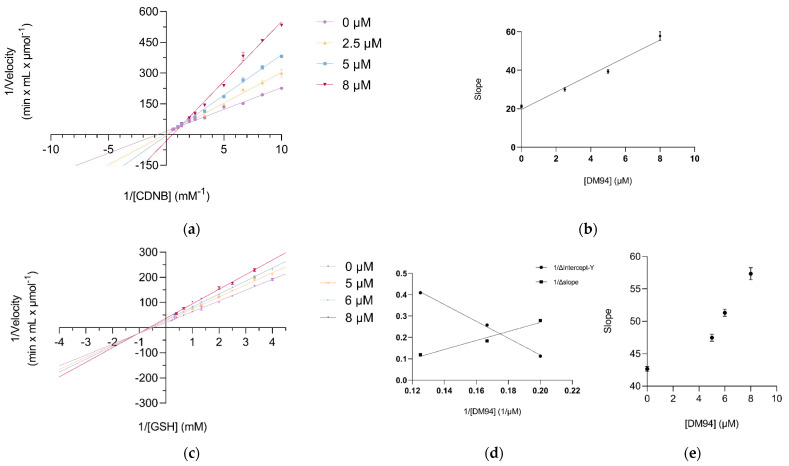
Kinetic inhibition analysis of *Cp*GST by compound DM94. (**a**) Lineweaver–Burk plots using CDNB as the variable substrate at fixed GSH concentration, with increasing concentrations of DM94 (0, 2.5, 5, and 8 μM). (**b**) Secondary plot of slopes from panel (**a**) as a function of DM94 concentration, used to calculate the inhibition constant (K_i_). (**c**) Lineweaver–Burk plots using GSH as the variable substrate at fixed CDNB concentration, with increasing concentrations of DM94 (0, 5, 6, and 8 μM). (**d**) Secondary plot of slopes from panel (**c**) as a function of DM94 concentration. (**e**) Tertiary plot showing 1/ΔIntercept-Y and 1/ΔSlope versus 1/[DM94], confirming mixed-type inhibition and enabling the determination of K_i_ and K_i_′ values.

Given its strong inhibitory activity and the absence of prior reports on its interaction with GSTs, the compound DM94 was selected for detailed kinetic inhibition studies to determine its inhibition constant (K_i_) and the mode of inhibition. The Lineweaver–Burk plots using CDNB as the variable substrate are shown in Figure 8a. The kinetic lines in the absence and presence of three different concentrations of DM94 intersected in the first quadrant, which is indicative of a mixed-type inhibition pattern. This suggests that DM94 is capable of binding to both the free enzyme and the enzyme–substrate (Enzyme-CDNB) complex, with respective inhibition constants K_i_ and K_i_′. A secondary plot of the slopes from the double reciprocal plots versus DM94 concentration (Figure 8b) displayed a linear relationship (R^2^ = 0.9838), confirming a purely mixed-type inhibition mechanism. The calculated inhibition constants were K_i_ = 4.35 ± 1.47 μM and K_i_′ = 3.20 ± 0.61 μM.

When GSH was used as the variable substrate, the Lineweaver–Burk plots again demonstrated mixed-type inhibition (Figure 8c). The corresponding secondary plot exhibited a nonlinear (parabolic) relationship (Figure 8d), indicating a partial mixed-type inhibition mechanism. The estimated inhibition constants were K_i_ = 5.04 ± 0.36 μM for the free enzyme and K_i_′ = 4.13 ± 0.68 μM for the enzyme–GSH complex (Figure 8e).

### 2.7. Therapeutic Implications

From a therapeutic standpoint, GSTs in protozoa offer several advantages as drug targets. First, their role in mitigating oxidative stress and metabolizing xenobiotics renders them essential under host-induced stress conditions. Second, significant structural divergence from mammalian GSTs allows for the development of parasite-selective inhibitors. Third, as shown in other protozoa, GSTs are implicated in resistance mechanisms, making them suitable for combination therapies aimed at sensitizing parasites to conventional drugs [59]. In light of these considerations and given the structural and functional divergence of *Cp*GST from human GST orthologs, which underscores its potential as a selective target with minimal off-target effects on host enzymes, *Cp*GST can be considered as a promising candidate for anti-cryptosporidial drug development. Its structural uniqueness, essential functional role, and druggability provide a solid foundation for future structure-based drug discovery efforts.

Prior biochemical studies have shown that *C. parvum* oocysts exhibit extremely limited antioxidant enzyme activity, with only low levels of superoxide dismutase (SOD) detected and no measurable activity of catalase, peroxidase, or classical GSTs [55]. These findings suggest that the GSTs encoded in the *C. parvum* genome may perform noncanonical or stress-induced roles, possibly activated only under specific environmental conditions. The absence of detectable GST activity in earlier studies may be attributed to the enzyme’s expression pattern or to its low specific activity, as demonstrated in the present study. Moreover, the role of GSTs in redox homeostasis must be considered in the broader context of Cryptosporidium’s redox biology. Recent work by Gabriele et al. (2025) has shown that *C. parvum* lacks authentic glutathione reductase and instead relies on a thioredoxin reductase (*Cp*TrxR)-based system to maintain glutathione in its reduced form [60]. *Cp*TrxR, a type II thioredoxin reductase with unique structural features, serves as a central redox regulator and is essential for parasite viability [60]. This suggests a functional integration between the thioredoxin and glutathione systems, with GSTs potentially participating downstream in detoxification and signaling pathways. Given that *Cp*TrxR is irreversibly inhibited by the gold-based drug auranofin, which also impairs GST-related pathways, this finding raises the possibility of redox-targeted combination therapies in cryptosporidiosis.

Although the present biochemical and inhibition data support *Cp*GST as a promising candidate for further investigation, its essentiality for *C. parvum* survival, infectivity, or life-cycle progression has not yet been experimentally demonstrated. Future studies should include genetic or functional validation approaches and parasite infectivity assays in relevant host cell models. Such studies will be necessary to determine the biological importance of *Cp*GST and to establish whether its inhibition can compromise parasite viability or infectivity.

## 3. Materials and Methods

### 3.1. Materials

Natural products (ellagic acid, (±)-taxifolin hydrate, gallic acid, (−)-epigallocatechin gallate, p-coumaric acid, (±)-naringenin, quercetin, curcumin, resveratrol, colchicine, polydatin, safranal, piperlongumine) were obtained from Sigma-Aldrich (St. Louis, MO, USA). The curcumin analogues were synthesized as previously reported [31]. Dimethyl sulfoxide (DMSO), acetone and ethanol were purchased from Scharlau (Barcelona, Spain). The antibiotics kanamycin and chloramphenicol were bought from AppliChem (Darmstadt, Germany). CDNB, reduced glutathione, cumene hydroperoxide and all other substrates were purchased from Sigma-Aldrich (St. Louis, MO, USA). The plasmid vector pET-28b bearing the 6-His tag from Novagen was employed for the subcloning of the *Cp*GST gene. PCR experiments were performed using the KAPA Taq polymerase from KAPA Biosystems (Wilmington, MA, USA) and Platinum™ Pfx from Invitrogen™ (Carlsbad, CA, USA). Moreover, CloneAmp™ HiFi PCR Premix polymerase from Clontech Laboratories, Inc. (Takara Bio, San Jose, CA, USA) was used in the subcloning reactions.

### 3.2. Methods

#### 3.2.1. Biocomputing-Based Structural Characterization of CpGST

Structural prediction of the *Cp*GST enzyme was performed using the I-TASSER server [61,62]. Among the generated models, the one with the highest confidence score (C-score = −0.13) was selected for further analysis. The predicted 3D structure was visualized and examined using UCSF Chimera 1.16. Functional domain analysis was carried out via the InterPro database [63], while homologous sequences were identified through BLASTp searches against the Protein Data Bank (PDB) and non-redundant protein sequences [64]. The UniProt database and the resulting hits were used to construct a phylogenetic tree using the iTOL web tool (version 7.6) [65]. Multiple sequence alignment of the retrieved homologs was conducted using Clustal Omega [66,67], and the results were graphically represented with ESPript [68]. The three-dimensional structure of the enzyme was also predicted using the AlphaFold2 server [69]. AlphaFold2 model quality was evaluated from the per-residue pLDDT traces, using the standard interpretation that pLDDT ≥ 90 indicates high accuracy, 70–90 indicates confident backbone placement, and <70 indicates flexible/low-confidence regions [70].

Molecular docking analysis was performed to investigate the putative binding mode of the best ligand DM57 within the predicted three-dimensional structure of *Cp*GST. The AlphaFold2-predicted structure of *Cp*GST was used as the receptor model. Molecular docking calculations were carried out using DiffDock-L [71] (run on the Neurosnap web server https://neurosnap.ai/, accessed on 9 July 2026). Docking evaluation was based on binding affinity, minimized affinity, minimized RMSD, and intramolecular energy. The best-ranked docking pose was selected based on the docking confidence score. Prediction of the ligand binding site and analysis of the enzyme–ligand complex were carried out using the ProteinsPlus web server (https://proteins.plus) [72].

#### 3.2.2. Molecular Cloning of the CpGST

The gene encoding a putative GST from *Cryptosporidium parvum* Iowa II (Accession No.: XP_628625.1) was identified through an extensive search of the NCBI database. The *Cp*GST gene was synthesized (GeneScript, Piscataway, NJ, USA) and codon-optimized for enhanced expression in *Escherichia coli*. PCR amplification was performed using the In-Fusion^®^ HD Cloning Kit (Takara Bio USA, Inc.) with the following primers:

Forward: 5′-AGAAGGAGATATACCATGATTGAATATCTAGAGCACAATATATC-3′.

Reverse: 5′-GTGGTGGTGGTGGTGACGGATGTACGGCTTTAAC-3′.

The PCR reaction (25 μL) contained 12.5 μL of CloneAmp™ HiFi PCR Premix (1X), 0.3 μM of each primer, 16 ng of DNA template and 9.5 μL of ddH_2_O. The amplification protocol included an initial denaturation at 98 °C for 4 min, followed by 35 cycles of denaturation at 98 °C for 10 s, annealing at 62 °C for 15 s, and extension at 72 °C for 10 s. A final extension step was carried out at 72 °C for 10 min. The resulting amplicon was cloned into the pET-28b expression vector (Lucigen, Middleton, WI, USA), which contains a C-terminal hexahistidine (6×His) tag. The resulting construct, pET-28b_CpGST_6×His, was initially used to transform *E. coli* Stellar™ competent cells. Plasmid DNA was then isolated using the NucleoSpin^®^ Plasmid kit (Macherey-Nagel, Düren, Germany), sequenced to confirm the correct insertion and the presence of the C-terminal 6×His tag, and subsequently used to transform a range of *E. coli* expression strains, including BL21 (DE3) pLysS, BL21 (DE3), BL21 OverExpress™ C41 (DE3), BL21 Rosetta™ 2 (DE3) pLysS, BL21 Shuffle^®^ T7 Express, and BL21 Tuner™ (DE3).

#### 3.2.3. Heterologous Expression and Purification of CpGST

High levels of heterologous expression of the pET-28b_*Cp*GST_6×His construct were achieved in *E. coli* Rosetta™ 2 (DE3) pLysS cells. The expression protocol was adapted with minor modifications from a previously established method [69]. Briefly, transformed cells were initially grown in LB medium at 37 °C for 16 h. A suitable volume of this pre-culture was then used to inoculate flasks containing 2×YT medium (1.6% *w*/*v* tryptone, 1% *w*/*v* yeast extract, and 0.5% *w*/*v* NaCl) supplemented with kanamycin (100 µg/mL) and chloramphenicol (34 µg/mL). When the optical density at 600 nm reached approximately 0.5–0.6, protein expression was induced by the addition of 1 mM IPTG, followed by incubation at 37 °C for 18–20 h. After expression, cells were harvested by centrifugation at 8000× *g* for 8 min, and the resulting pellet was resuspended in wash buffer (300 mM NaCl, 50 mM NaH_2_PO_4_, pH 8.0). Cell lysis was performed by sonication (6 cycles of 15 s at 60 Hz, 50 W), and the lysate was clarified by centrifugation at 12,000× *g* for 10 min for purification.

Recombinant *Cp*GST was purified by immobilized metal affinity chromatography (IMAC) using Ni^2+^-IDA Sepharose resin pre-equilibrated with wash buffer [70]. The column was washed, and the bound protein was sequentially eluted with buffers containing increasing concentrations of imidazole: 10 mL with 5 mM, 15 mL with 10 mM, and a final 5 mL fraction with 20 mM imidazole, all in 50 mM NaH_2_PO_4_ buffer containing 300 mM NaCl (pH 8.0). All purification steps were carried out at 4 °C. Eluted fractions were diluted with glycerol to a final concentration of 50% (*v*/*v*) and stored at −20 °C for further analysis.

#### 3.2.4. Kinetic Analysis

The kinetic characterization of the recombinant *Cp*GST enzyme was performed to determine its Michaelis–Menten parameters (K_m_, V_max_, k_cat_) using GSH and 1-chloro-2,4-dinitrobenzene (CDNB) as substrates. Enzyme activity assays were conducted based on established protocols with slight modifications [58,71]. Reactions were carried out in a final volume of 1 mL at 25 °C. For assays in which GSH served as the variable substrate, the reaction mixture contained 0.1 M KH_2_PO_4_ buffer (pH 6.5), a fixed concentration of CDNB, and varying concentrations of GSH (0–5.5 mM). Conversely, when CDNB was the variable substrate, the mixture contained the same buffer and CDNB concentrations ranging from 0 to 2.4 mM. In all assays, the enzyme was pre-incubated with GSH in the buffer for 5 min at 37 °C prior to the addition of CDNB to initiate the reaction. The kinetic parameters K_m_, V_max_ and k_cat_ were calculated by fitting the data to the Michaelis–Menten equation using GraphPad Prism software (version 9.3.1).

#### 3.2.5. Inhibitor Screening and Kinetic Inhibition Analysis

Selected natural products [ellagic acid, (±)-taxifolin hydrate, gallic acid, (−)-epigallocatechin gallate, p-coumaric acid, (±)-naringenin, quercetin, curcumin, resveratrol, colchicine, polydatin, safranal, piperlongumine] and the synthetic curcumin analogues were evaluated as potential inhibitors of *Cp*GST. Compounds were dissolved in DMSO to a final concentration of 100 μM. Inhibitory activity was assessed by measuring *Cp*GST catalysis using GSH and CDNB as substrates at final concentrations of 2.5 mM and 1 mM, respectively. The formation of the conjugated product was monitored at 340 nm for 120 s at 25 °C. Test compounds were added to the reaction mixture at a final concentration of 25 μM. Prior to initiating the reaction with CDNB, the enzyme was pre-incubated with GSH and the respective compound in buffer at 37 °C for 5 min. Compounds showing the greatest inhibitory effect were selected for IC_50_ determination. For this, enzymatic activity was measured in the presence of increasing concentrations of each inhibitor under the same assay conditions. All measurements were performed in triplicate, and appropriate controls and corrections were applied as needed. IC_50_ values were calculated by nonlinear regression using GraphPad Prism (version 9.3.1). Kinetic inhibition analysis was conducted to elucidate the mode of inhibition, following the methodology used for the initial kinetic characterization. The substrate concentration (GSH or CDNB) was varied while keeping the other constant, in the absence and presence of fixed concentrations of the inhibitor. All assays were conducted in triplicate. Kinetic data were analyzed using Lineweaver–Burk plots, and the inhibition type was determined based on the intersection patterns of the plotted lines. All enzymatic assays were performed in triplicate, and data are presented as mean ± standard deviation. Kinetic parameters and IC_50_ values were calculated by nonlinear regression analysis using GraphPad Prism. For inhibitor screening performed at a single compound concentration, the data were used for comparative ranking, and no pairwise statistical significance was inferred unless specifically indicated.

## 4. Conclusions

This study offers the first in-depth molecular and functional characterization of a GST from *Cryptosporidium parvum*, unveiling a structurally distinct enzyme with conserved catalytic residues and unique features that distinguish it from host homologs. The enzyme’s low substrate affinity, atypical structural elements, and evolutionary classification suggest a specialized role in the parasite’s biology. The identification of high affinity curcumin inhibitors opens new scaffolds for rational drug design. Furthermore, the parasite’s reliance on a thioredoxin-based redox system, as recently elucidated, may enhance the therapeutic relevance of GST inhibitors when used in combination with redox-disrupting agents. Future studies should assess the effects of DM46, DM57, and DM94 in *C. parvum*-infected cell culture models, including parasite growth/infectivity assays and host cell cytotoxicity evaluation, to help in advance *Cp*GST as a viable target in the fight against cryptosporidiosis.

## Figures and Tables

**Table 1:** Substrate specificity of purified recombinant *Cp*GST. Results represent the means of triplicate determinations with a variation of less than 5% in all cases.

Substrate	Specific Activity (U/mg)
CDNB (1-chloro-2.4 dinitrobenzene)	0.43
CuOOH (Cumene hydroperoxide)	0.25
tert-BuOOH Tert-butyl-hydroperoxide	0.01
Fluorodifen	0.18
DHA (Dehydroascorbate)	Undetectable
2-[2,3-Dichloro-4-(2-methylidenebutanoyl)phenoxy]acetic acid (Ethacrynic acid)	Undetectable
AITC (Allyl isothiocyanate)	Undetectable
PEITC (Phenethyl isothiocyanate)	Undetectable

**Table 2:** Kinetic parameters of the *Cp*GST enzyme determined using GSH and CDNB as variable substrates. Values represent the mean ± standard deviation (SD) from three independent experiments.

Kinetic Parameters	Substrate	*Cp*GST
K_m_ (mM)	GSH	3.10 ± 0.40
K_m_ (mM)	CDNB	1.75 ± 0.22
k_cat_ (s^−1^)		4.46 ± 0.06

**Table 3:** Screening of the inhibitory potential of selected natural products and curcumin analogues against *Cp*GST enzyme activity. Compounds were tested at a final concentration of 25 μM. Results are expressed as the percentage inhibition relative to the control (no inhibitor) and represent the mean ± SD of three independent experiments. ND: Not determined due to the absence of inhibition.

Natural Products			
Molecular Structure	Enzyme Inhibition (%)	Molecular Structure	Enzyme Inhibition (%)
(±)-Taxifolin hydrate 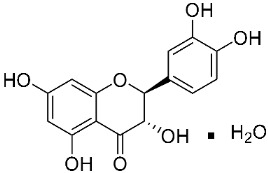	47.6 ± 0.1	Polydatin 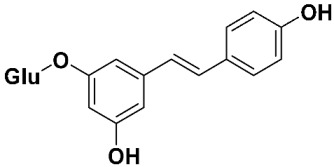	34.7 ± 1.1
(±)-Naringenin 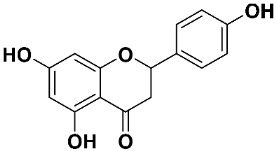	43.5 ± 1.0	Gallic acid 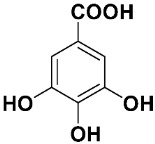	34.3 ± 2.6
(−)Epigallocatechin gallate 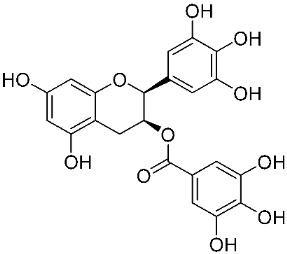	40.7 ± 1.7	p-Coumaric acid 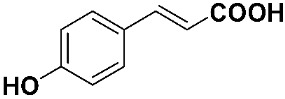	25.8 ± 1.4
Quercetin 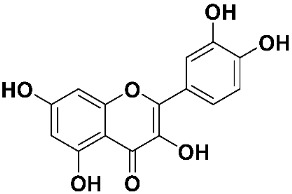	38.1 ± 0.1	Resveratrol 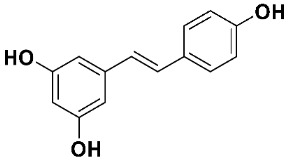	15.4 ± 1.0
Curcumin 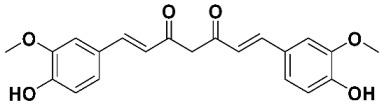	37.6 ± 2.0	Colchicine 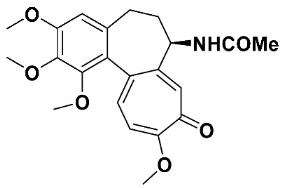	8.7 ± 1.1
Ellagic acid 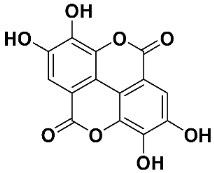	ND	Safranal 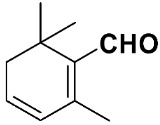	ND
Piperlongumine 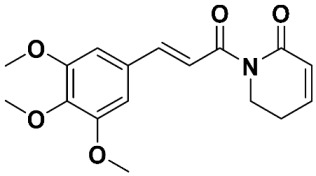	ND		
Curcumin analogues			
DM94 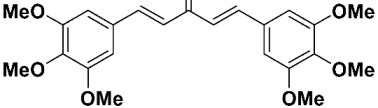	64.9 ± 2.4	DM109 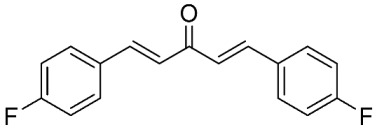	16.3 ± 0.9
DM46 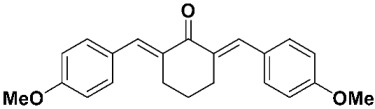	65.2 ± 1.2	DM95 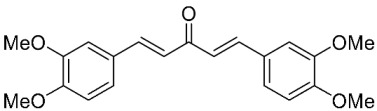	15.9 ± 0.7
DM57 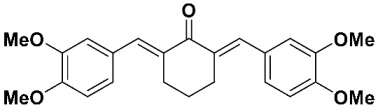	62.3 ± 1.5	DM96 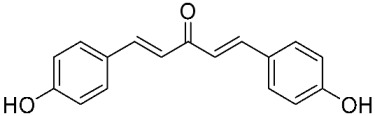	15.5 ± 0.9
MS238 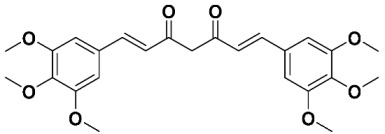	31.6 ± 1.3	DM151 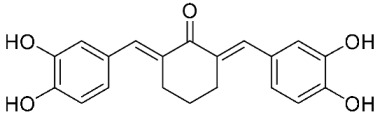	14.2 ± 2.9
DM16 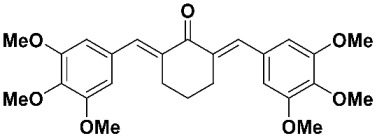	29.5 ± 2.3	DM62 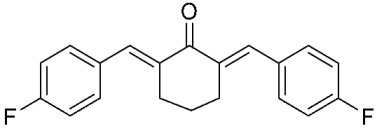	13.4 ± 0.8
DM236 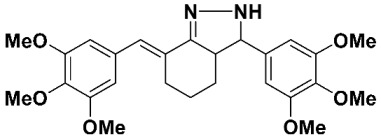	13.0 ± 1.4	DM101 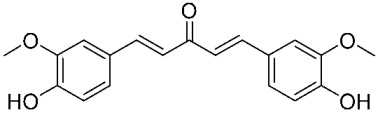	4.5 ± 1.9
DM148 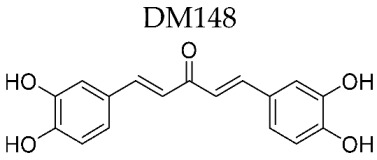	11.8 ± 2.0	DM15 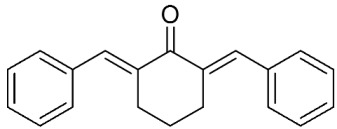	ND
DM100 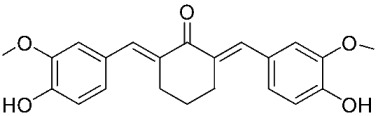	7.2 ± 2.5		

ND: Not determined due to the absence of inhibition.

**Table 4:** IC_50_ values of the tested compounds DM46, DM57, and DM94 determined by dose–response kinetic inhibition assays against *Cp*GST enzyme activity.

Tested Compound	IC_50_ Values (μM)
DM46	17.6 ± 0.6
DM57	11.8 ± 0.5
DM94	16.8 ± 0.5

## Data Availability

The original contributions presented in this study are included in the article/Supplementary Material. Further inquiries can be directed to the corresponding author.

## Supplementary Materials

The following supporting information can be downloaded at: https://www.mdpi.com/article/10.3390/ph19071106/s1, Figure S1: Structural comparison and AlphaFold2 confidence analysis of *Cp*GST.

## Abbreviations

The following abbreviations are used in this manuscript.

CDNB	1-Chloro-2,4-dinitrobenzene
*Cp*TrxR	*Cryptosporidium parvum* Thioredoxin Reductase
GSH	Glutathione (reduced form)
GST	Glutathione transferase
*Cp*GST	*Cryptosporidium parvum* GST
H-site	Hydrophobic substrate-binding site of GST
IC_50_	Half-maximal Inhibitory Concentration
IPTG	Isopropyl β-D-1-thiogalactopyranoside
LB	Luria–Bertani medium
Ni^2+^-IDA	Nickel–iminodiacetic acid (used in affinity chromatography)
ORF	Open Reading Frame
SOD	Superoxide Dismutase
Trx	Thioredoxin
TrxR	Thioredoxin Reductase
