# Supplementary material for: Functional Characterization and Inhibition Analysis of a Glutathione Transferase from Cryptosporidium parvum: A Potential Target for Antiparasitic Drug Development
Source: Pharmaceuticals (Basel). 2026 Jul 17;19(7):1106. doi: 10.3390/ph19071106 (PMC13414986; doi:10.3390/ph19071106)
Supplement: Supplementary file 1 [file pharmaceuticals-19-01106-s001.zip › pharmaceuticals-4405906-supplementary.pdf]

a

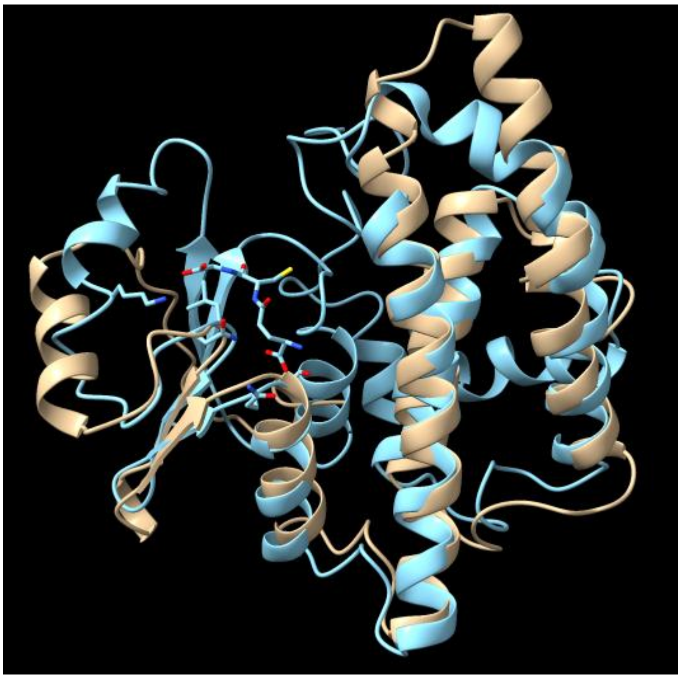

b

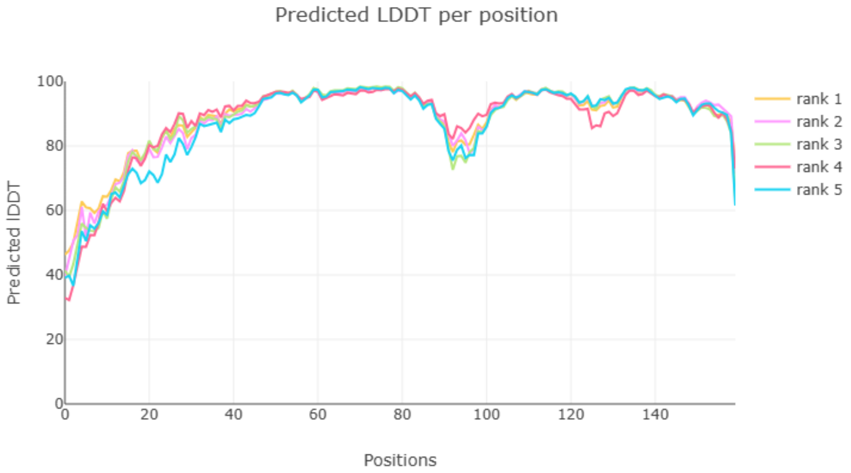

Figure S1. Structural comparison and AlphaFold2 confidence analysis of *Cp*GST. (a) Structural superposition of the AlphaFold2-predicted *Cp*GST model with the mu-class GST from *Fasciola hepatica* in complex with GSH (PDB ID: 1FHE). *Cp*GST is shown in light brown, while *Fasciola hepatica* GST and bound GSH are shown in light blue. (b) Per-residue confidence analysis of the AlphaFold2-predicted *Cp*GST structures, assessed using the predicted Local Distance Difference Test (pLDDT). The pLDDT profiles of the five ranked AlphaFold2 models are overlaid as colored traces. pLDDT values indicate local model confidence, with scores >90 corresponding to very high confidence, 70–90 to good confidence, 50–70 to low confidence, and <50 to regions that may be disordered or poorly resolved.
